# Challenges in Improving Adherence to Diet and Drug Treatment in Hypercholesterolemia Patients

**DOI:** 10.3390/ijerph20105878

**Published:** 2023-05-19

**Authors:** Francesco Baratta, Francesco Angelico, Maria Del Ben

**Affiliations:** Department of Clinical Internal, Anaesthesiologic and Cardiovascular Sciences, Sapienza University of Rome, 00161 Rome, Italy

**Keywords:** diet, statins, lipid-lowering therapy, adherence to treatment

## Abstract

Poor adherence to chronic disease treatment may seriously compromise the effectiveness of therapy, characterizing itself as a critical element for the population’s health, both from the point of view of quality of life and health economics. The causes of low adherence are many and can depend on the patient, the physician and the healthcare system. Low adherence to dietary recommendations and lipid-lowering drug therapy for hypercholesterolemia is a widespread phenomenon that may strongly limit the great advantages of serum lipid reduction strategies in primary and secondary cardiovascular prevention. Many patients discontinue treatment, and adherence decreases with time. Increasing therapeutic adherence can have a much greater impact on the health of the population than any other therapeutic advance. There are numerous strategies to increase therapy adherence according to behavior change theories. They concern the doctor and the patient. Some must be implemented at the time of prescription, others later during the follow-up. The active role of the patient in the therapeutic decision and the shared definition of LDL cholesterol targets are of paramount importance. The aim of this narrative review is to summarize evidence on current levels of adherence to lipid-lowering strategies, the causes of the lack of adequate adherence and possible physician-applicable strategies to improve it.

## 1. Introduction

The primary and secondary coronary events prevention strategies obtained through the reduction of LDL cholesterol have represented one of the most important achievements of medicine over the last 30 years. Numerous primary and secondary prevention clinical trials conducted with diet and drugs have convincingly demonstrated that LDL cholesterol levels are related to cardiovascular risk in a dose-dependent manner [1,2]. This confirms the “LDL hypothesis” of atherosclerosis based on the concept that elevated LDL cholesterol values are a causal factor in atherosclerosis development.

The current lipid-lowering therapies represent one of the major advances of modern medicine. They play a central role in reducing morbidity and mortality due to cardiovascular, cerebrovascular and peripheral vascular diseases [3,4,5]. Statins (HMG-CoA reductase inhibitors) are the first-line drugs in lipid-lowering therapy, as clearly indicated by the guidelines [1,6,7]. More recently, similar results have been observed in clinical trials with lipid-lowering combination therapy with Ezetimibe and PCSK9 inhibitors [1]. Particularly significant results have been obtained in primary prevention in patients with familial hypercholesterolemia and other genetically based dyslipidemia and in secondary prevention in those with previous acute coronary syndrome. In both conditions, the use of lipid-lowering therapy causes a significant reduction in cardiovascular morbidity and mortality.

However, the beneficial effect observed in large randomized clinical trials can be translated into “real-life” only in the presence of good adherence to the prescribed therapy [8]. Regarding lipid-lowering therapy, adherence observed in the real world is much lower than that described in clinical trials with either statin monotherapy or combination therapy and tends to decrease over time. Moreover, lipid-lowering therapy’s low adherence is associated with a lower efficacy of cardiovascular prevention [9,10].

Low adherence to treatment is now recognized as a major public health problem, which can affect morbidity, mortality and healthcare costs [8]. Poor adherence to dietary and pharmacological prescriptions is the main cause of the treatment’s low efficacy and represents a critical aspect of being carefully evaluated. It is estimated that approximately 50% of patients do not correctly take the drugs prescribed for long-term therapies [11,12]. Moreover, some data fix the level of compliance to lipid-lowering therapy at a lower level. In an observational study conducted in Croatia, only 35% of patients met >70% of the prescribed statin dose, and just half (51%) fully complied [13].

In the case of chronic diseases, poor adherence to therapy seriously compromises the effectiveness of their treatment, characterizing itself as a critical element for the population’s health, compromising patients’ quality of life and affecting the health economy budget [14,15]. Therefore, it is important to know the causes of therapies’ poor adherence and identify behavioral and organizational interventions to achieve better adherence to prescriptions. Increasing medication adherence can have a greater impact on population health than the introduction of new therapies [16].

Similarly, non-adherence to international dietary recommendations [17] is a critical phenomenon that reduces the efficacy of cardiovascular prevention, and it is related to the increase in all-cause mortality [18]. For these reasons, the latest guidelines editions emphasized the importance of dietary and lifestyle changes to reduce cardiovascular risk, suggesting the best dietary options and strategies to improve the patient’s adherence to this kind of prescription [1,6,19].

It is, therefore, essential to strengthen the culture of patients and physicians on the importance of good adherence to dietary recommendations and drug therapy.

## 2. Review Aim and Literature Search Strategy

The aim of this narrative review is to summarize the evidence on current levels of adherence to lipid-lowering strategies, the causes of the lack of adequate adherence and possible strategies physicians can apply to improve it. We retrieved the literature from PubMed databases published up to 28 February 2023. The following keywords were used: “Statin adherence” and lipids; “Diet adherence” and lipids; “Dietary adherence” and lipids; “Behavior change theory” and lipids; “Behaviour change theory” and lipids; “Social Cognitive Theory “ and lipids; “Social Ecological Model” and lipids; and “Planned Behavior Theory” and lipids. After excluding non-English language and unavailable manuscripts, a total number of 399 manuscripts were found. Reviews, randomized clinical trials and observational studies focusing on adherence to statin and diet to improve lipids were retrieved. References from relatively important manuscripts were also retrieved.

## 3. Adherence: The Definition

Generally, adherence to prescription is assessed by means of compliance, adherence and persistence (Table 1). Compliance is the degree to which a patient follows a prescription. The term suggests that the patient is following accurately, but passively, what the doctor prescribed. Adherence is the degree to which the patient agrees with the indications. The term underlines the patient’s participation in the dietary-therapeutic decision. Non-adherence can be divided into primary non-adherence (i.e., failure to initiate therapy) and secondary non-adherence (i.e., discontinuation of therapy). Secondary non-adherence can further be classified as failure to take medication according to prescription (dose and frequency of therapy) or premature discontinuation of the medication. In most studies, good adherence is defined as patients who took at least 80% of the prescribed drug dose or who took the drug on at least 80% of the scheduled days [20].

Comprehensive assessment of causes of low adherence related to the patient, the physician and the health system can help predict the course of therapy. The most predictive factors are the possible side effects of the therapy, the absence of disease symptoms, the complexity of the therapeutic regimen, the presence of depression and cognitive impairment of the patient and the bad physician–patient relationships. These will lead to inadequate follow-up and the patient’s loss of confidence in the therapy [20].

## 4. Barriers to Good Adherence

There are several causes of therapy low adherence or discontinuation, some are obvious and easily identifiable, and others are subtler and more elusive and specific to a given treatment/drug. Most of the causes can be classified into three main categories: patient-related causes, prescriber-related ones and factors related to the healthcare system’s organization (Table 2). Very often, there are interactions between the various causes of poor adherence [20,21,22].

The main patient-related causes include basic characteristics such as low socio-cultural level, instability of the family environment, the presence of comorbidities and practical difficulties in carrying out the therapy. Sometimes the patient decides not to be treated and comes to deny the presence of his disease or consider it untreatable. However, very often, voluntary attitudes prevail, such as the lack of understanding of one’s pathology and its seriousness, the presence of negative experiences with previous therapies, and above all, the lack of confidence in the effectiveness of the therapy and in the competence of the physician. In addition, defiant attitudes towards the physician or the disease can also occur. Another reason for low adherence is disappointment in not having obtained the expected benefits. By contrast, in the presence of positive effects, patients could abandon the treatment early. Recently, a new cause of low adherence to therapy is represented by the amount of information available online and the risk of its misinterpretation by patients who self-interpret them without referring physician support. In addition, complex therapeutic regimens based on polytherapy with multiple daily pill intakes can lead to poor adherence to the therapeutic schedule. Finally, for some drugs, particularly statins, possible adverse effects, such as muscular toxicity, may also be relevant [20,21,22]. The main physician-related causes include the prescription of multi-therapies and complex regimens, the failure to apply the guideline recommendations [1,6,7] and the lack of interest in assessing patient adherence. Another important cause may be the incomplete explanation to the patient of his clinical condition, the possible benefits of the prescribed therapy and of any adverse effects. Finally, the difficulty of accessing the physician, his specialization, the conflicting information given by several physicians and the prescriptions with substitutions between equivalent drugs can be other causes of reduced adherence [20,21,22].

The main health system-related causes of poor adherence are the consequence of the need to reduce management costs and the complexity of administrative procedures to access outpatient facilities. It follows a reduction in doctor–patient interaction times, resulting in the impossibility of assessing adherence and activating initiatives to increase it. Finally, the last cause may be the high costs of some therapies [20,21,22].

## 5. Adherence to Dietary Recommendations

A good adherence to a Mediterranean-type low-fat diet and healthful lifestyle is associated with lower serum cholesterol levels and reduced odds of having hypercholesterolemia. Therefore, current guidelines recommend initial dietary counseling by physicians for most patients with hypercholesterolemia and referral to a registered dietitian for those with persistent hypercholesterolemia [1,6,19]. The main dietary recommendation is to minimize the consumption of saturated fats to lower LDL-C, as well as other therapeutic dietary options, such as increasing plant stanols/sterols and increasing viscous fiber [1].

However, despite the proven beneficial effects of dietary recommendations on controlling hypercholesterolemia, compliance to dietary recommendations is difficult to achieve, patient adherence remains sub-optimal and few patients follow dietary recommendations. Indeed, less than 50% of United States adults follow dietary recommendations and the need to improve patient adherence is a major issue in controlling serum cholesterol. Causes of low adherence to dietary recommendations may vary from person to person. Among the reasons, obstacles and motivations for poor adherence to cholesterol-lowering diets, the low perception of risk and the poor patient education about hypercholesterolemia play a major role. Moreover, many patients are not convinced about diet efficacy and declare no need for information or help to conform to a diet. Finally, patients’ traditions, culture and dietary preferences, as well as the bad physician–patient relationship, may also be associated with poor dietary compliance [23].

In a large survey conducted in France, when patients (whether medically treated or untreated) were asked about the reasons for not complying with a prescribed diet, the four top answers were ‘already having satisfactory food habits’ (34.7%), ‘unwillingness to suffer nutritional deprivation’ (33.3%), ‘difficulties to conciliate a diet with family life’ (27.8%) and ‘taking cholesterol-lowering drugs’ (22.2%) [24]. In a recent study, factors of diet non-adherence to AHA diet in hypercholesterolemic patients (52.35% of total variance) included “situational barriers and gathering”, “takeaways and eating out”, “psychological factors”, “false beliefs and food habits”, “lack of motivation”, “enjoy eating and difficulty resisting the temptation” and “satisfaction with previous food habits” [23]. Barriers may also relate to doctors’ lack of conviction in the efficacy/usefulness of diet and low confidence in the ability to maintain dietary modifications long-term [23].

## 6. Adherence to Lipid-Lowering Drug Therapy

Many patients discontinue lipid-lowering therapy or fail to reach the therapeutic targets indicated by the guidelines [1,6,7], and the adherence to the therapeutic schedule in the real world is much lower than that described in clinical trials.

Recently, a low percentage of subjects at target for LDL-C was demonstrated by the DA VINCI study, which enrolled 5888 patients on lipid-lowering therapy in 18 European countries between June 2017 and November 2018 [25]. In both primary and secondary prevention, moderate-intensity statin monotherapy was the most frequent therapy in all risk categories. Compared with the LDL-C targets indicated in the 2016 guidelines [26] for the different risk categories, only 54% of the subjects were on target. This proportion was 63%, 75%, 63% and 39% in low-, moderate-, high- and very-high-risk subjects, respectively. As expected, the LDL target was more easily achieved with high-intensity statin therapy alone or in combination with Ezetimibe or PCSK9 inhibitors. The proportion of those on target fell to 19% in very high-risk subjects when the LDL cholesterol targets proposed in the 2019 guidelines [1] were taken into consideration, which, as known, have been further reduced.

Similar data were obtained in the United States in a study on 4106 patients with a previous coronary event. Less than 50% were treated according to guideline recommendations for reasons due to the patient (rejection or side effects) or physician (stable and well-controlled LDL-C). Approximately 30% were not on statin therapy, mainly due to a doctor’s decision, and 22% took a lower dose of the drug than indicated by the guidelines due to the appearance of side effects [27]. These results demonstrate the presence of a significant gap between the guidelines [1,6] and clinical practice in achieving the LDL-C targets and suggest the need for greater use of combination therapies with high-intensity statins. However, in clinical practice, failure to achieve targets can also be due to low adherence to therapy, which can be largely sub-optimal.

We should consider that about a quarter of patients discontinue therapy within 6 months [28] and a half of the patients within the first year [29]. However, adherence to therapy decreases over time. This was well demonstrated in a large study conducted in two Boston hospitals investigating the leading causes of discontinuation of statin therapy in over 100,000 subjects. About half of the subjects had discontinued therapy for a period of at least 12 months, and the main reasons reported by the patient were “no longer needed, no longer prescribed, discontinued by insurance or too expensive”. Statin-related adverse events were documented in only 17% of patients, and of these, after rechallenge with a statin, over 90% were still on therapy after 1 year [29].

An interesting study was conducted on a large cohort of elderly subjects to whom statins were prescribed after acute coronary syndrome. A good adherence was observed in about 70% of patients. The adherence was lower in patients treated with coronary revascularization compared to those treated with medical therapy. The authors conclude that the absence of symptoms after performing coronary revascularization may explain the patient’s perception of a lower need for medical therapy with statins [30]. Moreover, it should be considered that the benefits of the therapy are not immediately visible to the patient, while the appearance of any side effects can occur early. Very low adherence to statin therapy was also observed in China, both in patients in primary prevention and in secondary prevention. Most discontinuations occurred within the first three months after a statin prescription. Adherence was about 30% after the first month and decreased strongly in the second and third months to reach only 10% after the first three months [31]. Suboptimal adherence was also observed in Florence, Italy, in a cohort of subjects who had received a first statin prescription. At 1 year, adherence was low in 26% of subjects, low/intermediate in 16.2%, high/intermediate in 19.7% and high in 41.1%. Subjects with low adherence were younger and had fewer comorbidities [32]. Similar data were observed in Lombardy in a large series of subjects who had been prescribed statins. Approximately 60% of subjects had low or very low adherence to therapy, and only 20% showed good adherence. Again, the less adherent subjects were younger and had fewer comorbidities [33].

In a further study of 90,000 subjects starting statin therapy, adherence to prior chronic medications better predicted adherence to therapy at 1 year than patient demographics and clinical variables [34]. These results were confirmed in a retrospective cohort study conducted on 22,925 patients enrolled in the Texas-based Medicare Advantage plan, in which the ability to adhere to previous background therapy with ACE inhibitors, ARBs and oral antidiabetics was associated with adherence to statin therapy 1 year after their first prescription [35]. Both studies suggest that prior good adherence to chronic therapies predicts and improves adherence to statins when they are first prescribed. Greater adherence has been described in cohorts of patients with diabetes and dyslipidemia, where more than 70% adhered to statin therapy and approximately 40% achieved guideline-recommended LDL-C target values [1,36,37].

Recently, a large study conducted in Canada evaluated the effect of age and gender agreement between patients and doctors on adherence one year after a first statin prescription. The study concludes that age and gender concordance are not significant predictors of good adherence [38]. Finally, a telephone survey was conducted within Kaiser Permanente Northern California (KPNC) to assess concerns and barriers to statin prescribing [39]. The most reported concerns about statins preferred to lower cholesterol with lifestyle changes (66%), disliking the drugs in general (59%) and liver or kidney problems (31%); the most common reason for taking a lower dosage was having difficulty remembering to take statins (9%). Subjects with greater awareness of elevated cardiovascular risk were less likely to be non-adherent [39].

Recent data showed a small but significant improvement in statin adherence over the last two decades among patients in secondary prevention (from 73.1% to 76.2%) and in those in primary prevention at high risk (from 57.9% to 60.1%). By contrast, no improvement was observed in those in primary prevention without a history of type 2 diabetes (from 57.6% to 58.5%) [40]. Again, the same research identified black/Hispanic, female gender, young age (less than 54 years), low adherence to other chronic therapies and number of pills taken daily as the strongest predictors of non-adherence to statin therapy [40].

The concern about the use of statins may reduce their use in some conditions associated with a high cardiovascular risk. Non-alcoholic fatty liver disease (NAFLD) associates with high cardiovascular risk [41]. Data from the PLINIO study demonstrated that, despite the high cardiovascular risk, only 44% of NAFLD patients with an indication for lipid-lowering therapy took statins [42]. Instead, a recent meta-analysis, resuming data from 22 studies, demonstrated that statins are safe and efficient in NAFLD patients [43].

Statin intolerance due to muscle symptoms is a possible cause of low adherence or therapy discontinuation. However, it is infrequent and often appears only with very high dosages and not with all statins. Statin intolerance was retrospectively evaluated in 16,717 patients with dyslipidemia being treated in 23 Italian Lipid Clinics. The prevalence of muscle symptoms was 9.6%; however, after statin dechallenge (interruption of treatment) and/or rechallenge (modification or resumption of statin therapy), there was a reappearance of muscle symptoms in only 3.01% of the entire series [44]. Recently a meta-analysis conducted on over 4 million subjects treated with statins confirmed a prevalence of statin intolerance of 9.1% [45]. Prevalence was significantly higher in cohort studies (17%) than that observed in randomized clinical trials (4.9%). Statin intolerance was higher among women, in subjects with diabetes, hypothyroidism, chronic liver and kidney disease and in those taking antiarrhythmic drugs and calcium channel blockers, thus confirming the importance of careful clinical evaluation of the patient. A further study demonstrated that adherence to therapy could improve if patient care is intensified and if a rechallenge is made with the same or with another statin [46].

## 7. Physician Role in Behavior Health Theories Application and Possible Strategies to Improve Adherence

It is very important that clinicians know that behavioral health theories could be helpful in achieving better patient adherence to lipid-lowering prescriptions [47]. Despite the use of behavioral health theories for lipids management being less investigated than for other cardiovascular risk indicators, such as blood pressure (5% vs. 46%), the use of Social Cognitive Theory and the Social Ecological Mode is the most used and investigated theories to improve patients’ behavior on lipids [47].

The Social Cognitive Theory (SCT) by Albert Bandura [48] defines the importance of self-regulated behaviors through the self-efficacy evaluation based on regular or familial performance and on social support monitoring. Another key point of this theory is the definition of an outcome expectation, which incentives patients’ prescription adherence. The application of SCT has proven to be efficacy in favoring lifestyle change and improving nutritional aspects and physical activity [49].

The Social Ecological Model (SEM) provides a framework for developing multidimensional preventive strategies at the intrapersonal, interpersonal, organizational, community and policy levels [50] to prevent and modify environmental influences on behavior at micro-, meso-, exo- and macro-system levels [51]. The Heart of New Ulm Project, a population-based project with health care, community and workplace interventions targeting multiple levels of the SEM, demonstrates the efficacy of this model in improving the use of lipid-lowering therapies and patients’ adherence to it [52].

The efficacy of the Planned Behavior Theory (PBT), another behavioral health theory, has been demonstrated in familial hypercholesterolemia patients [53]. The PBT proposes intentions as the most important predictor of behavior. Intentions are a function of sets of personal-, social- and control-related beliefs [54]. In addition, PBT could be successfully integrated with the self-efficacy process from SCT [55].

**Figure 1:**
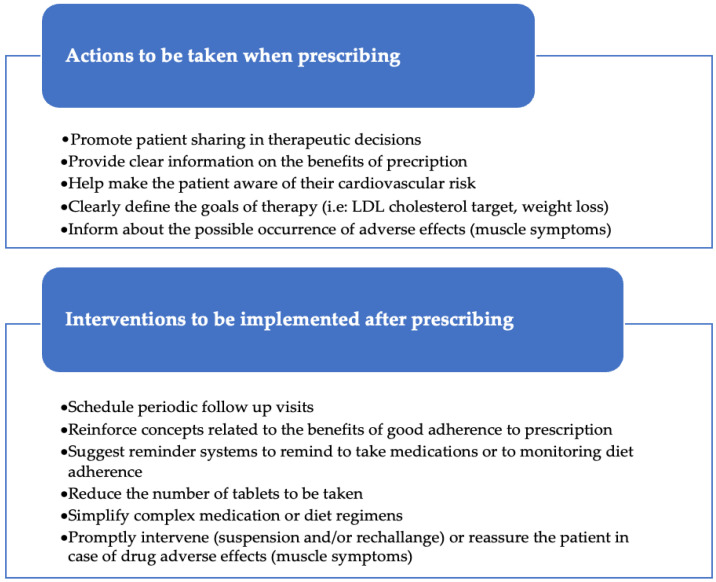
Physician-applicable actions to improve patients’ adherence to lipid-lowering therapy.

Figure 1 reports that some of the actions, extrapolated from all the discussed theories, could be applied by physicians to improve the patient’s adherence to their prescriptions (Figure 1) [8,9,22].

First of all, it is very important that the doctor carefully checks if the patient has understood his/her health problem and the importance of the dietary and drug prescriptions. Poor patient awareness of the health problem reduces goal setting and compliance to therapies whose usefulness was not known [56]. Then, another crucial condition for obtaining good adherence is the sharing between the patient and the doctor of the therapeutic decisions at the time of the first dietary or pharmacological prescription. The shared decision-making process improves patients’ adherence and goal achievement [57,58]. Patients must have an active role in the therapeutic decision [59]. This helps them understand the reason for the treatment and the benefits it can bring, including the fact that good adherence to treatment can help prolong their life and reduce cardiovascular risk. These concepts should be extended to the patient’s family environment, which will thus be able to support adherence to therapy. Patients should acquire an awareness of their own cardiovascular risk in order to adjust their lifestyle and maintain good adherence to therapy over time. Furthermore, the doctor has to share with the patient the final objectives of the therapy and clearly define the LDL cholesterol targets to be achieved in relation to the individual level of cardiovascular risk. All these initiatives will contribute to producing a valid rationale to maintain good adherence to therapeutic prescriptions [22].

After therapy prescription, it must be implemented a careful monitoring that allows the identification of any problems or adverse effects to be promptly addressed with the active participation of the patient [60]. This can be achieved through frequent follow-up visits, telephone contacts, the simplification of complex therapeutic regimens, the collaboration of social and health personnel and the activation of reminders also using digital tools [61]. At this stage, the involvement of dietitians and digital tools can also be beneficial in improving dietary adherence among hypercholesterolemic patients [62].

As demonstrated in other settings, a trained healthcare professional, even other than the physician, who guides patients through the follow-up steps and goal achievements, can promote patients’ adherence and persistence to prescriptions [63].

Furthermore, a diary to record medication intake can be an invaluable tool for increasing medication adherence by serving as both a motivating tool and a visible reminder for medication intake [64]. This action, taken together with a simpler therapeutic regime using single-pill fixed combination therapy, may help improve prescription adherence [65].

Finally, intensifying patient care and simplifying the administrative procedures for access to follow-up visits prolong the doctor–patient interaction times and actively motivate the patient to continue with the therapy or to make any changes to it. In addition, it will also be useful to increase the knowledge of both the doctor and the patient on the importance of maximizing lipid-lowering therapy, particularly in those at higher risk [60]. Finally, in the case of statin therapy, it will be essential to intervene promptly in case of intolerance onset by implementing a short period of suspension of therapy and rechallenge with the same statin or a different one (or a lower dose) [20].

Despite promising data suggesting a positive role of human technology in diet and drug adherence monitoring and improvement [61,62,66], there is a wide gap in the knowledge of the efficacy of the application of new digital tools to improve patient adherence and persistence to both dietary and drugs. It will be useful to investigate how the periodic administration of electronically delivered food diaries and questionnaires might improve patients’ adherence to dietary prescriptions. Similarly, the improvement of drug adherence using a digital app tracking drug prescription, purchase and intake, integrating health system data with the user-friendly app for patients use, should be investigated more. Finally, in our opinion, digital systems could deeply and easily integrate different levels of interventions (intrapersonal, interpersonal, organizational, community and policy levels) required by the above-discussed behavior health theories.

Finally, we must consider that multifaceted and tailored interventions are more efficacy than single-focus and generalized ones [67].

## 8. Conclusions

Good adherence to prescribed treatment is more effective for health than the introduction of new therapies. However, adherence to dietary and drug prescriptions tends to decrease over time, decreasing the efficacy of therapeutic strategies. Most of the barriers to good adherence are physician-related, and more efforts by scientific societies are needed to improve physician consciousness on their role in adherence improvement strategies. In addition, scientific society should perform a stronger lobbying action on health system players to obtain the application of systematic strategies to reduce non-adherence. Finally, in our opinion, the poor knowledge of how the new human technology could favor both drugs and dietary adherence represents, to date, the most important lack on the matter and new research focused on this aspect is needed.

## Figures and Tables

**Table 1:** Definition of compliance, adherence and persistence to dietary recommendations or drug regimen.

Terminology	Definition
Compliance	Compliance is the degree to which patients, when on a diet or taking a drug that has been prescribed to them, respects dietary recommendations or the dosage indications and times of intake indicated by the prescribing doctor. The term suggests that the patient is following accurately, but passively, what the doctor prescribed. Compliance is measured over a period and reported as a percentage.
Adherence	Adherence is the degree to which the patient on a diet or taking a drug complies, after having shared them, with the indications of a healthcare professional. The term underlines the patient’s participation in the medical-therapeutic decision. It is the product of a patient-caregiver relationship that is based on respect, active participation and collaboration, not coercion or manipulation. Non-adherence can be divided into primary non-adherence (i.e., failure to initiate therapy) and secondary non-adherence (i.e., discontinuation of therapy).
Persistence	Persistence refers to the act of continuing the treatment for the prescribed duration. It is the time between the start and the end of a prescribed dietary or drug treatment. Persistence may be reported as a dichotomous variable measured at the end of a predefined period, considering patients as being “persistent” or “nonpersistent”.

**Table 2:** Main causes of poor adherence to lipid-lowering therapy.

**Patient-related**	Denial of the disease and/or its possibility of being cured Presence of comorbidities and polytherapy Lack of knowledge of the possible benefits of the therapy Poor confidence in the efficacy of the therapy or in the competence of the doctor Low socio-cultural level, instability of the family environment, advanced age Absence of clear goals of therapy (LDL cholesterol target) Poor involvement in decisions about treatment Negative experiences with previous therapies Poor communication between healthcare professionals and patients Misinformation from social media
**Physician-related**	Little time is dedicated to informing the patient about the possible benefits of the therapy Poor explanation of the pros and cons of proposed treatment Little effort is placed into assessing adherence to therapy Prescription of multi-therapies and complex therapeutic regimens Conflicting information from doctors with different specialties Little consideration of how to make information accessible and understandable Little possibility is given to the patient to be involved in making decisions about prescribed treatment Poor understanding of the patient’s knowledge, beliefs and concerns about treatment Failure to apply the recommendations of the guidelines Confusion and misinformation about the prescription
**Health system-related**	Long times to book specialist outpatient visits Little time to devote to the patient during the visit Rotation of doctors in the same practice The complexity of the bureaucratic/administrative aspects High costs of some therapies Poor cooperation from health professionals such as nurses and pharmacists

## Data Availability

Not applicable.
